# Generalized morphoea in the setting of combined immune checkpoint inhibitor therapy for metastatic melanoma: A case report

**DOI:** 10.1097/MD.0000000000025513

**Published:** 2021-04-23

**Authors:** Ewan A. Langan, Kaja Budner, Detlef Zillikens, Patrick Terheyden

**Affiliations:** a Department of Dermatology, University of Lübeck, Lübeck, Germany; b Dermatological Sciences, University of Manchester, Manchester, UK.

**Keywords:** case report, immunotherapy, melanoma, morphoea

## Abstract

**Rationale:**

Immune checkpoint inhibition has dramatically altered the therapeutic landscape in the treatment of a range of locally advanced and metastatic skin cancers. In particular, the treatment of metastatic melanoma with combined anti-programmed cell death protein 1 (anti-PD1) and anti-cytotoxic T-lymphocyte-associated protein 4 (anti-CTLA4) antagonists has resulted in median 5-year survival rates of over 50%. However, combined immune checkpoint inhibitor therapy frequently results in the development of immune-related adverse events (irAE) which can be severe and life-threatening. While the typical irAEs, namely colitis, thyroiditis, and hepatitis are well recognized, cutaneous irAEs are varied and can be difficult to accurately diagnose.

**Patient concerns:**

A 61-year-old female with metastatic melanoma presented with widespread indurated, waxy skin changes, and weight loss following combined anti-PD1 and anti-CTLA4 immunotherapy.

**Diagnoses:**

Generalized morphea in the setting of combined immunotherapy.

**Interventions:**

Dexamethasone pulse therapy (100 mg i.v. over 3 days) was combined with topical therapy (clobetasone propionate ointment) and physiotherapy. Four cycles of dexamethasone pulse therapy, at 4 weekly intervals, led to an improvement in the skin changes, accompanied by increased mobility. However, the changes did not resolve completely.

**Outcome:**

Staging examinations revealed progressive melanoma brain metastases and despite 2 further cycles of combined anti-PD1 and anti-CTLA4 immunotherapy followed by 1.5 cycles of Fotemustine, the patient died 22 months after the development of the scleroderma-like skin changes.

**Lessons:**

Cutaneous irAEs are varied in nature and severity. Sclerotic skin changes are rare, but unlike cutaneous irAEs related to immune checkpoint inhibitor therapy, they are often refractory to standard treatment with systemic corticosteroids. Clinicians should be aware of immunotherapy-related scleroderma to prompt dermatological evaluation to facilitate early recognition and initiate treatment. Administration of systemic immunosuppression should be carefully balanced against the risk of promoting melanoma progression.

## 1 Introduction

Combined immune checkpoint inhibitor therapy (anti-programmed cell death protein 1 [anti-PD1] and anti-cytotoxic T-lymphocyte-associated protein 4 [anti-CTLA4] monoclonal antibodies) often results in the development of immune-related adverse events (irAE) which can be severe, persistent, and life-threatening. While the typical irAEs, namely colitis, thyroiditis, and hepatitis are well recognized, cutaneous irAEs vary in nature and severity and can be difficult to accurately diagnose.

While most clinicians are aware of the classical irAEs, the significance of cutaneous irAEs may be less well recognized and even under-reported, due to their typically mild and diverse nature. Immune checkpoint inhibitor therapy can induce maculo-papular, psoriasiform, eczematous, lichenoid, and even bullous skin changes. While these dermatoses are often mild, associated with pruritus and respond to topical steroid therapy, severe cutaneous irAEs in association with immune checkpoint inhibitor therapy, including autoimmune blistering diseases and life-threatening toxic epidermal necrolysis, have been reported.^[1]^ Moreover, it is important to bear in mind that immune checkpoint-mediated dermatoses may first present months after the initiation of treatment.^[2]^ Therefore dermatological assessment is important to facilitate early diagnosis and initiate treatment, especially in the case of severe cutaneous toxicities.^[3]^

Here we report a case of scleroderma-like skin changes is the setting of immune checkpoint inhibitor treatment of metastatic melanoma to draw attention to a rare cutaneous adverse event associated with immunotherapy and to illustrate that its management often requires systemic treatment to facilitate clinical improvement and to minimize the risk of permanent skin changes and disabling contractures.

## 2 Case report

We report a 61-year-old female patient who first presented 9 years ago with a melanoma on the left forearm (pT2a). Although the sentinel lymph node biopsy was negative, the patient developed loco-regional metastases 2 months later, despite adjuvant low dose Interferon-alpha-2a treatment. These were treated surgically (excision of the in-transit metastases and axillary lymph node dissection). A BRAF mutation was absent. After a further 2 months, staging examinations revealed the presence of pulmonary and hilar lymph node metastases. Treatment with dacarbazine (1000 mg/m^2^) was then administered every 3 weeks and resulted in radiologically stable disease. In an attempt to achieve a tumor-free status, that patient underwent a mediastinal lymph node dissection and removal of the lung metastasis in the right lower lobe. Although the lymph nodes were tumor free, the pulmonary melanoma metastasis reached the resection margins and therefore 3 weekly dacarbazine (1000 mg/m^2^) treatment was recommenced. After 16 cycles in total the patient developed further pulmonary metastases and 3 cycles of ipilimumab (3 mg/kg) were administered. Ipilimumab was discontinued due to the development of severe immune-mediated colitis (Grade 3, Common Terminology Criteria for Adverse Events [CTCAE]) requiring treatment with systemic corticosteroids and infliximab. Subsequent treatment (dacarbazine, surgery, and radiotherapy) failed to halt disease progression. Immunotherapy (pembrolizumab) was commenced (2 mg/kg every 3 weeks) but withdrawn after 23 cycles due to intracerebral disease progression.

Despite the history of immune-mediated colitis, the decision was made to initiate combined ipilimumab (3 mg/kg) and nivolumab (1 mg/kg) immunotherapy. Two weeks after the first administration, the patient developed an immune-mediated thyrotoxic crisis (CTCAE Grade 3) which was successfully managed with propranolol and thiamazol. After an 8-week treatment interruption, the second cycle of ipilimumab (3 mg/kg) and nivolumab (1 mg/kg) was administered. The following staging examinations revealed a partial intracranial response and stable extracranial disease. On this basis, and in light of the history of immune-mediated colitis, the decision was made to temporarily interrupt immunotherapy treatment. Ten months after the initial combined immunotherapy, the patient developed skin changes on the abdomen, breasts, and limbs which were neither painful nor pruritic. The patient had also developed profound cachexia and had lost 14 kg in weight over several months. With the exception of type II diabetes, there was no personal or family history of either autoimmune or rheumatological diseases.

**Figure 1:**
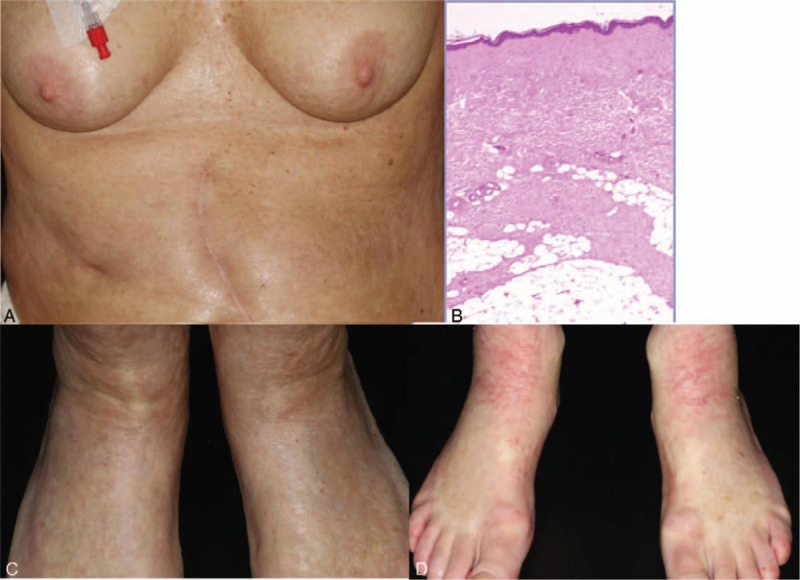
The clinical and histopathological presentation. Waxy, infiltrated sclerotic plaques on the trunk (A) and feet (B). Histology of a skin biopsy from the arm revealed fibrosis in the upper dermis with an interstitial lymphocytic infiltrate (C). There were thickened septae in the subcutaneous fat with focal homogenization of the collagen fibres. An eosinophilic infiltrate was absent. The clinical findings and mobility responded partially to pulsed dexamethasone therapy (D) but the sclerosis and superficial eczematous changes remained.

On examination, there were sclerotic plaques on the abdomen and breasts, with sparing of the peri-areolar skin. In addition, there were erythematous and skin colored papules and plaques, which partly coalesced on the feet, lower legs, and forearms (Fig. 1A and B). The face and hands were not affected. On palpation there was hardening of the skin, resulting in reduced mobility.

Routine blood tests showed an eosinophila (1.14 × 10^9^/L, normal range 0.02–0.5 × 10^9^/L) and elevated C-reactive protein levels (34 mg/L, normal range <5 mg/L). The anti-nuclear antibody (ANA) titre was 1:160 and rheumatoid factor was negative. A skin biopsy revealed fibrotic changes and a mild panniculitis with thickened septae in the subcutaneous fat (Fig. 1C). There was no evidence of an eosinophilic fasciitis. Esophageal manometry revealed impaired esophageal mobility with evidence of achalasia. On the basis of the characteristic skin changes, the histological findings and the laboratory investigations, a diagnosis of generalized morphoea was made and treatment with dexamethasone pulse therapy (100 mg i.v. over 3 days) was initiated. This was combined with topical therapy (clobetasone propionate ointment) and physiotherapy. Four cycles of dexamethasone pulse therapy led to an improvement in the skin changes, accompanied by increased mobility (Fig. 1D), but the changes did not fully resolve. Systemic corticosteroid treatment was withdrawn due to the development of progressive melanoma brain metastases. Despite 2 further cycles of combined anti-PD1 (nivolumab 1 mg/kg) and anti-CTLA4 (ipilimumab 3 mg/kg) immunotherapy 3 weeks apart, followed by 1.5 cycles of Fotemustine, the patient died 22 months after the development of the scleroderma-like skin changes.

## 3 Discussion

Scleroderma-like skin changes associated with immunotherapy are rare. Tjarks et al^[4]^ reported scleroderma-like skin changes in a 61-year-old male after 16 cycles of nivolumab treatment for renal cell carcinoma. In fact, the skin changes, which failed to respond to cessation of nivolumab, required treatment with both systemic glucocorticoids and mycophenolate mofetil. Recurrence of morphea has also been reported during treatment with nivolumab, although in this case the skin changes occurred only 2 months into treatment.^[5]^ Pembrolizumab has also been associated with the development of scleroderma-like skin changes in 3 patients with metastatic melanoma, occurring between 5 and 13 cycles of therapy.^[6,7]^ In each case, systemic glucocorticoid treatment was insufficient to arrest the skin changes and additional immunosuppressants (mycophenolate mofetil, cyclophosphamide, and or infliximab) and immunomodulatory (immunoglobulins, hydroxychloroquine) agents were required.^[6,7]^ Most recently, 2 further cases of limited cutaneous systemic sclerosis have been reported during pembrolizumab therapy for non-small cell lung cancer, albeit both had evidence of pre-existing connective tissue disease.^[8]^ Nevertheless, the disease flare resulted in the development of a scleroderma renal crisis in one of the patients and both required treatment with prednisolone and cyclophosphamide. In contrast, Cho et al reported a case of nivolumab-associated “scleroderma-like syndrome” which responded to systemic glucocorticoids and cessation of the checkpoint inhibitor.^[9]^ Based on Terrier et al's review of the WHO pharmacovigilance database, VigiBase, 35 cases of scleroderma during immune checkpoint inhibition therapy have been reported.^[8]^

The mechanism underlying immune checkpoint therapy-induced scleroderma is unclear. However, transforming growth factor beta (TGFβ) may be implicated, given its role in the pathogenesis of systemic sclerosis and that PD-1 inhibition results in TGFβ activation.^[7]^ Furthermore, circulating levels of soluble PD-1 reportedly contribute to the development of systemic sclerosis.^[10]^

To our knowledge, we report the first case of generalized morphea associated with eosinophila following combined ipilimumab and nivolumab immunotherapy. It is interesting to note that the use of anti-TNFα treatment for the treatment of immune-mediated colitis did not prevent the patient from developing sclerotic skin changes. We cannot determine whether prior treatment with ipilimumab monotherapy, and subsequently pembrolizumab, may have predisposed the patient to the development of this rare irAE. Given that cutaneous irAEs, namely vitiligo, are actually associated with an improved response to immune checkpoint inhibition, the relationship between scleroderma-like skin changes and overall survival should be established. In any case, given that generalized morphoea may be triggered and/or exacerbated by immune checkpoint inhibition, and that these changes are often refractory to the standard management of irAEs, that is, systemic glucocorticoids; clinicians should be aware of this cutaneous irAE facilitate early recognition and initiate treatment. Any decision to administer potent systemic immunosuppression should be carefully balanced against the risk of promoting melanoma progression and is best taken in a multi-disciplinary context.

## Author contributions

**Conceptualization:** Ewan A. Langan, Kaja Budner, Patrick Terheyden.

**Formal analysis:** Kaja Budner, Patrick Terheyden.

**Investigation:** Kaja Budner.

**Resources:** Detlef Zillikens, Patrick Terheyden.

**Supervision:** Patrick Terheyden.

**Writing – original draft:** Ewan A. Langan.

**Writing – review & editing:** Ewan A. Langan, Kaja Budner, Detlef Zillikens, Patrick Terheyden.
